# When the body changes faster than the brain: a narrative and theoretical review of body schema updating after bariatric surgery

**DOI:** 10.3389/fnut.2026.1874945

**Published:** 2026-08-11

**Authors:** Beatrice Fava, Chiara Spiezia, Claudia Di Rosa, Laura Dalla Ragione, Yeganeh Manon Khazrai

**Affiliations:** 1 Research Unit of Food Science and Human Nutrition, Department of Sciences and Biotechnologies, Università Campus Bio-Medico di Roma, Roma, Italy; 2 Operative Research Unit of Nutrition and Prevention, Fondazione Policlinico Universitario Campus Bio-Medico, Roma, Italy

**Keywords:** bariatric surgery, body dissatisfaction, body image, body schema, embodied cognition, implicit body representation, obesity

## Abstract

As indicated by the guidelines of the National Institutes of Health (NIH) and the Italian Society of Obesity Surgery and Metabolic Diseases (SICOB), bariatric surgery is the treatment of choice for severe obesity due to its effectiveness in achieving long-term weight loss and improving associated comorbidities. Many individuals with obesity exhibit profound body dissatisfaction, which often represents a primary motivation for seeking bariatric surgery, sometimes outweighing health-related reasons. Following surgery, a significant reduction in body weight is typically observed; however, perceived body image does not always change in parallel with physical transformation. Some individuals experience difficulties adapting to bodily changes, with a persistent perception of themselves as people with obesity despite substantial weight loss. This phenomenon is typically interpreted in terms of body image disturbances; however, it may also indirectly point to difficulties in updating body representations, potentially involving body schema mechanisms, although this has not yet been directly investigated in bariatric populations. Given the limited number of studies on body schema in this context, this article provides a narrative and theory-driven review of body representation following bariatric surgery. Specifically, it integrates findings from body image research, obesity studies, and embodied cognition to explore whether body schema updating may contribute to the mismatch between physical and perceived body. This interpretation remains hypothetical and highlights a critical gap in the literature, namely the lack of direct empirical investigations of body schema in bariatric populations. Further research is needed to clarify its role in post-operative adaptation and its implications for psychological and behavioural outcomes.

## 1 Introduction

Bariatric surgery is the treatment of choice for severe obesity, leading to long-term weight loss and the improvement or resolution of comorbidities (1, 2). Despite the well-documented health benefits, many individuals affected by obesity opt for bariatric surgery primarily because of body dissatisfaction. In some cases, aesthetic concerns outweigh medical reasons, as rapid weight loss and visible changes in body shape contribute to improved self-image, self-esteem, and the alleviation of psychological problems associated with excess weight (3).

However, the significant body transformation induced by bariatric surgery does not necessarily imply a corresponding update of body representations, either at the level of body image or body schema. Body image and body schema represent distinct but interacting systems of body representation. Body image is the mental representation of one’s body – including its shape, size, and the personal and emotional experience related to it – and constitutes a long-term and conscious perception of the self (4). On the other hand, body schema refers to the automatic and implicit experience of one’s body and is therefore connected to the anatomical-functional characteristics of the nervous system, such that one is aware of one’s body both at rest and in motion (5).

Interestingly, despite substantial weight loss, some individuals continue to identify themselves as people with obesity and exhibit persistent body dissatisfaction (6). Although the persistent perception of oneself as a person with obesity is generally interpreted as an alteration of body image, the potential contribution of body schema and its updating mechanisms to post-bariatric adaptation remains largely unexplored. This review aims to explore whether and how mechanisms related to body schema updating may contribute to the mismatch between physical changes and body perception after bariatric surgery, by integrating indirect evidence from psychological and neurocognitive literature. Given the absence of direct empirical investigations of body schema in bariatric populations, the present model should be considered a theoretical framework that generates testable hypotheses rather than an evidence-based explanation of post-bariatric adaptation.

## 2 Methods

This narrative review was conducted following the principles of the Scale for the Assessment of Narrative Review Articles (SANRA). A non-systematic literature search was performed in PubMed, Scopus, and Google Scholar up to May 2025 using the following keywords and their combinations: “body schema,” “body image,” “implicit body representation,” “embodied cognition,” “body dissatisfaction,” “obesity,” and “bariatric surgery.” Eligible publications included empirical studies, reviews, and theoretical papers relevant to body representation, obesity, and bariatric surgery, whereas studies not related to body representation were excluded. Given the limited number of studies directly investigating body schema in bariatric populations, evidence from related fields, including embodied cognition, sensorimotor representation, and body representation research, was also considered. Study selection was guided by conceptual relevance and theoretical contribution to the objectives of the review rather than by predefined systematic criteria. The identified literature was narratively synthesised to integrate findings from body image research, obesity studies, and neurocognitive models of body representation, thereby developing a theoretical framework for understanding body schema updating after bariatric surgery.

## 3 Body schema

### 3.1 Historical origins of the body schema

**Figure 1:**
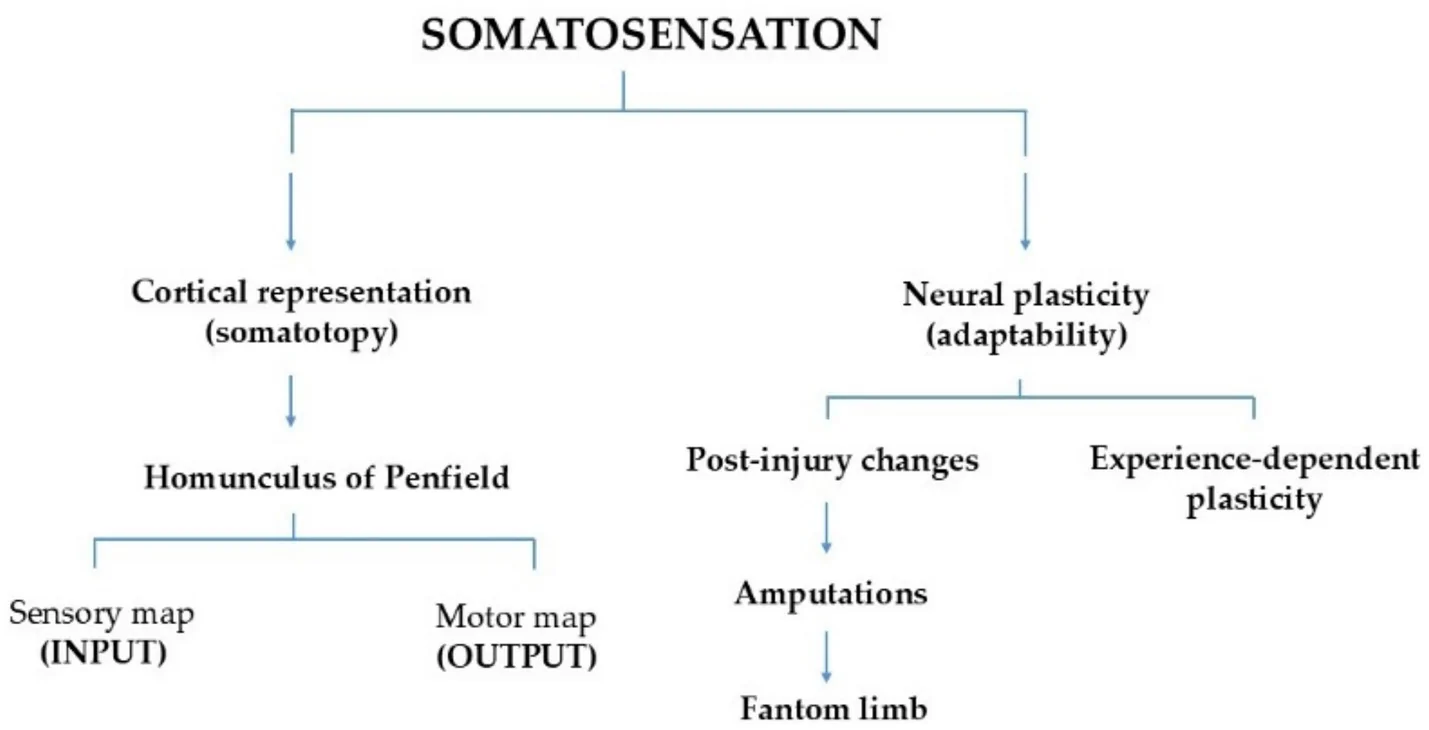
Body schema according to Penfield.

The concept of body schema emerged from early neurological observations aimed at explaining alterations in body awareness rather than normal body functioning. One of the most influential phenomena in this context was the *phantom limb* phenomenon described by Parè (7), in which amputated patients continued to experience sensations or movements of the absent limb. This suggested that bodily experience does not simply reflect the physical state of the body, but depends on centrally organised neural representations that can persist despite the loss of peripheral input. Within this clinical framework, Bonnier introduced the idea of a systematic representation of the body that supports spatial orientation and action, emphasising the functional role of body representations in the organisation of perception and movement (8). Pick argued that the body schema is based on mental images generated by tactile, kinesthetic, and, in particular, visual inputs. These images create a spatial representation of the body that develops over time, serving as an essential element for the awareness of one’s corporeality (9). This view was further developed by Head, who conceptualised the body schema as a dynamic construct derived from the continuous integration of proprioceptive, tactile, and vestibular signals (10). Building on these early concepts, Penfield’s neurosurgical observations provided direct evidence for the cortical basis of body representations, demonstrating that stimulation of specific cortical regions could induce localised body sensations, while revealing the plastic and experience-dependent nature of these maps in the human brain (11) (Figure 1).

Together, these early contributions converged on the view of the body schema as an implicit, multisensory representation grounded in neural processes and essential for the coherent experience and control of the body in space.

### 3.2 Body schema: functional organisation, sensorimotor integration, and plasticity

The body schema is a functional neural representation that emerges from the continuous integration of somatosensory, motor, and spatial information. Early experimental evidence suggested that somatosensory functions play a central role not only in the formation of the body schema but also in the perception of body size. Lautenbacher et al. (12) demonstrated that alterations in somatosensory processing contribute more to inaccuracies in body size estimation than affective or cognitive factors such as eating behaviour, body dissatisfaction, depressive symptoms, or emotional disorders (12). This supports the idea that distorted perceptions of body size mainly reflect changes in sensorimotor integration rather than psychological disorders.

From a functional perspective, body-centred perceptual attention is a key mechanism that allows body signals to reach cortical processing levels and guide intentional action. As Le Boulch highlighted, this form of attention allows individuals to go beyond automatic motor execution and act intentionally, situating the body in relation to the surrounding environment (13). Essentially, body perception is not limited to the physical boundaries of the body itself, but extends to the peripersonal space, i.e., the immediate space in which the body can interact with objects and other people. Through continuous interaction with the outside world, the neural systems involved in body representation, movement planning, and spatial processing cooperate to generate a unified sense of the self as a body acting in space (14) (Table 1).

**Table 1:** Main differences between body image and body schema.

Feature	Body image	Body schema
Nature	Conscious	Unconscious
Main components	Neuropsychological Emotional Socio-cultural	Neurological Sensorimotor Cortical processing

Experimental research conducted over the past few decades has provided substantial evidence that both body schema and peripersonal space are highly plastic and dynamically modulated by sensorimotor experience. Contemporary paradigms using virtual environments and tool-use tasks have demonstrated that consistent sensorimotor feedback can rapidly remap internal representations of the body. In a study conducted by D’Angelo et al. (15), participants controlled a virtual hand using an infrared motion capture system under synchronous and asynchronous feedback conditions. Only synchronous visual-motor feedback induced a strong sense of agency, leading to measurable changes in perceived forearm length and an expansion of peripersonal space. In contrast, asynchronous feedback did not induce such changes, highlighting the critical role of sensorimotor coherence in updating body representations (15).

Similarly, tool-use studies have consistently shown that the active and goal-directed use of external objects can temporarily extend the perceived boundaries of the body. Sposito et al. (16) demonstrated that using a tool influences the perceived length of the arm, suggesting that the tool becomes functionally integrated into the body schema (16). These findings suggest that body awareness is not a fixed property but a dynamic process shaped by continuous interactions between the body and the environment (17, 18).

Despite this well-documented plasticity, the body schema cannot be considered an infinitely malleable or unconditionally updatable construct. As early as 1998, Anita Witt Mitchell proposed that the body schema should not be considered a stable construct. It can change in response to alterations in body structure during growth, to different interactions with the environment, or to various sensory inputs. These factors can enhance or deteriorate the perception of the body schema and may lead to the creation of a mental image of the body that may or may not align with the actual body structure (19). This view underscores the idea that plasticity does not necessarily guarantee accurate or adaptive updating.

Repetto and Riva (20) further emphasised the reciprocal relationship between mind and body, highlighting the body as the primary medium through which individuals engage with the world (20). Taken together, these findings indicate that although body schema is fundamentally dynamic and plastic, its updating depends on specific conditions, including consistent sensorimotor input and active engagement with the environment. When these conditions are disrupted, profound body changes may not translate into corresponding updates of internal body representations. This observation is particularly relevant when considering situations in which the body undergoes rapid and substantial transformations, raising critical questions about the limits of body schema plasticity and providing the basis for the distinction between body schema and body image discussed in the following section.

## 4 Body image

### 4.1 Definition and foundational theories

The Austrian psychiatrist and psychoanalyst Paul Ferdinand Schilder is considered the founding father of current theories on body image development. He defined body image as the image of one’s body in one’s mind, that is, the way the body appears to oneself. According to his theory, body image is not static over one’s lifetime but is subject to continuous change (21).

Subsequently, the philosopher Gallagher (22) suggested that body image could be considered the conscious awareness of one’s body, encompassing perceptual, cognitive, and emotional dimensions (22).

Subsequently, the psychologist Peter D. Slade offered a more precise description of body image, asserting that it consists of a perceptual component (how people perceive the size and shape of their body) and an attitudinal component (what people think and know about their body). The latter component is considered more variable and may include an affective component, which reflects the feelings people have towards their body and the behaviours they engage in, such as dietary habits or physical activity levels (4).

### 4.2 Conscious-socio-emotional nature of body image

The representation of body image originates in an individual’s inner world and is constructed over time through the social meanings assigned to the body, the education received, and behavioural models. At the same time, body image is also shaped by the sensations and perceptions arising from the body, although emotional factors appear to play a predominant role in this process (23).

Body image can thus be considered a multidimensional construct that encompasses people’s conscious perception of their physical self, including the thoughts and feelings that stem from this perception. An individual’s body image develops and is maintained through complex interactions among socio-cultural, neuropsychological, and cognitive factors, thus influencing the behaviour (Table 1).

Body image can also be considered an element of personal identity and can be described as the subjective image individuals have of their bodies. An individual’s body image can relate to anthropometric measurements, body shape, and body form. In particular, it can reflect the level of satisfaction with one’s body or specific parts of it. Overall, it represents how we think, feel, perceive, and behave in relation to our body (24).

Based on this multidimensional concept, body dissatisfaction assumes a key role as an expression of body image, especially in obesity, as discussed in the following section.

## 5 Body dissatisfaction in obesity

Body dissatisfaction refers to the cognitive and affective experience of negatively evaluating one’s physical appearance, including body shape and weight (25). It refers to the discrepancy between the perceived body image and the ideal body image (26). This condition involves negative thoughts and feelings toward the body and is associated not only with eating disorders but also with elevated levels of depression, low self-esteem, and reduced quality of life (27).

As originally described by Stunkard and Mendelson (28), individuals with obesity often perceive their bodies as grotesque or repulsive in the eyes of others. These experiences foster a cyclical relationship between body dissatisfaction, depressive mood, and reduced self-esteem. Such dynamics appear to be particularly pronounced in individuals who experienced obesity during childhood or adolescence.

Among individuals with obesity, factors predisposing them to body image dissatisfaction may include stigma associated with obesity, the presence of eating disorders, dysfunctional eating behaviours, depression, and low self-esteem. People with obesity and body image disturbances often experience significant concern about their body image, believing that their physical appearance negatively impacts their self-worth (29, 30).

Stigmatising experiences and the internalisation of weight bias contribute to persistent dissatisfaction with body image, as well as to a distorted perception of one’s own body. Furthermore, many people with obesity report feelings of dissatisfaction so intense that they often describe feeling “trapped” in bodies that they perceive as excessively large (31, 32).

Body dissatisfaction in people with overweight or obesity can increase psychological distress, which may in turn intensify their efforts to restrict food intake or engage in excessive physical activity, potentially exacerbating the cycle of discomfort and frustration (33).

Within this representational framework, characterised by body dissatisfaction in individuals with obesity and a distorted perception of their own body, bariatric surgery emerges as one of the most frequently requested options, not only for weight loss but also for improving quality of life. It is often perceived as an intervention that promises immediate and lasting physical change.

### 5.1 Body dissatisfaction as a motivational driver for bariatric surgery

Makarawung et al. (34) showed that low evaluations of overall appearance represent a significant motivating factor for seeking bariatric surgery, highlighting the relevance of body image concerns beyond purely metabolic considerations (34).

Pearl et al. further demonstrated that the majority of patients seeking bariatric surgery report high levels of body dissatisfaction and a strong desire to alter specific body areas, such as the abdomen, legs, and arms. These motivations are often accompanied by unrealistic expectations regarding the extent of weight loss and the degree of bodily transformation achievable through surgery (35).

This perspective is further supported by a prospective study conducted among 210 bariatric surgery candidates, in which the authors showed that patients chose bariatric surgery not only to lose weight and improve obesity-related symptoms, but also to enhance body image and address pre-surgical body dissatisfaction (36).

Other publications have emphasised the central role of body image in the bariatric surgery process. Bertoletti et al. highlighted the importance of assessing body image perception before surgery, noting that candidates for bariatric procedures often have a distorted body image and aesthetic motivations that significantly influence surgical expectations. Consequently, the authors emphasised the need for a comprehensive assessment of body image as part of multidisciplinary care to identify unrealistic expectations and potential distortions of body image (37).

Finally, a comprehensive review by Ivezaj and Grilo (38) confirmed that negative body image is highly prevalent among individuals seeking bariatric surgery and is associated with adverse psychosocial outcomes. Although bariatric surgery appears to improve some aspects of body image, the authors highlight considerable heterogeneity among studies and a lack of systematic investigation into the multidimensional nature of body image throughout the bariatric process.

Taken together, these findings indicate that while health-related considerations are central to the decision to undergo bariatric surgery, body dissatisfaction represents a key driver of patients’ motivations and expectations.

Recent evidence has further highlighted the importance of psychological characteristics in bariatric surgery candidates and their role in post-bariatric adaptation. Factors such as body dissatisfaction, depressive symptoms, emotional dysregulation, unrealistic expectations, and maladaptive coping strategies may influence not only the decision to undergo surgery but also postoperative adaptation and long-term outcomes. In addition, weight loss following bariatric surgery has been associated with improvements in body image, self-esteem, and quality of life, highlighting the close relationship between physical changes and psychological well-being. However, despite these overall improvements, considerable individual variability persists, suggesting that successful adaptation to bodily change may depend on factors extending beyond weight loss alone, including the integration of new body-related representations. These findings indicate that psychological variables should be considered alongside physical factors when evaluating patients and planning postoperative support, as they may affect an individual’s ability to integrate bodily changes and adjust to their new body condition (39, 40).

## 6 Rapid morphological change and limits of body schema updating: a biobehavioural and neurocognitive perspective

### 6.1 Relationship between body image and body schema: why this distinction matters in bariatric surgery

The distinction between *body image* and *body schema* constitutes the conceptual foundation of the theoretical framework proposed in this review, as these two constructs describe distinct, although closely interconnected, levels of body representation. Body image refers to the conscious representation of the body, encompassing the perceptual, cognitive, affective, and evaluative processes through which individuals perceive and appraise their physical appearance (4, 22, 23). In contrast, body schema refers to an implicit sensorimotor representation that continuously integrates proprioceptive, tactile, vestibular, visual, and motor information to support posture, movement, and interactions with the surrounding environment (14, 41). Whereas body image represents how the body is consciously perceived and experienced, body schema reflects how the body functions as an acting organism within the environment.

This distinction is also reflected in the experimental paradigms used to investigate these constructs. Body image is typically assessed using self-report questionnaires, psychometric instruments that evaluate conscious body perception and attitudes toward one’s body. In contrast, because body schema operates primarily at an implicit level, it is investigated through experimental paradigms assessing sensorimotor integration, including body illusion paradigms, proprioceptive tasks, virtual reality protocols, and studies of peripersonal space (15, 16).

Although both body image and body schema are dynamic and plastic representations, they appear to update through different mechanisms and timescales. Body image can change relatively rapidly in response to visible bodily modifications or cognitive and emotional influences (23), whereas body schema depends on the gradual integration of multisensory and motor experience (10, 18).

It is important to emphasise that plasticity should not be interpreted as synonymous with unlimited adaptability. As proposed by Witt Mitchell (19), the body schema undergoes continuous modification throughout life in response to physical growth, interactions with the environment, and sensory experiences; however, these processes do not necessarily result in an accurate or adaptive update of the body representation. Consequently, substantial changes in body morphology may occur without a complete recalibration of the internal sensorimotor model of the body.

Although conceptually distinct, body image and body schema continuously interact and may mutually influence one another (42). Nevertheless, because they reflect different levels of body representation and rely, at least in part, on distinct neural systems, changes in explicit body representations do not necessarily imply a corresponding updating of implicit sensorimotor representations (41).

This distinction is particularly relevant in the context of bariatric surgery. Although numerous studies have documented improvements in body image following substantial weight loss, this alone does not adequately explain why profound morphological changes may coexist with the persistent subjective experience of inhabiting the pre-operative body. The concept of body schema provides an additional explanatory perspective by focusing on the updating of implicit sensorimotor representations. If body schema requires prolonged sensorimotor experience to recalibrate internal body models, the rapid morphological changes induced by bariatric surgery may temporarily outpace this recalibration, resulting in a mismatch between the objectively changed body and its internal sensorimotor representation. This potential discrepancy constitutes the theoretical premise of the present review and becomes relevant in light of the findings reported by Perdue et al. (43, 44), who, using the *Evolving Self-View* questionnaire, observed that many patients continued to identify themselves as people with obesity despite substantial postoperative weight loss.

Taken together, these observations raise the possibility that rapid morphological changes may not be accompanied by an equally rapid updating of implicit body representations. This hypothesis constitutes the conceptual starting point for the theoretical framework developed in the following section.

### 6.2 Rapid morphological change and limits of body schema updating: a biobehavioural and neurocognitive perspective

Although, as discussed in the previous sections, body image—the explicit representation of the body involving conscious evaluations of body size, shape, and appearance (45)—is strongly influenced by body dissatisfaction and represents a central motivation for undergoing bariatric surgery, available evidence indicates that such dissatisfaction does not automatically resolve following the substantial weight loss achieved through the procedure. In particular, several studies employing different designs and assessment tools show that, despite improvements in anthropometric parameters and overall health status, many patients may continue to report high levels of body dissatisfaction in the post-operative period (see Table 2). This suggests that objective bodily change does not necessarily translate into an improvement in how the body is perceived and evaluated.

**Table 2:** Studies that assessed body perception in the context of bariatric surgery.

References	Aims	Sample	Psychometric questionnaires	Results
Perdue et al. (43)	Exploring the experience of body image adaptation in bariatric patients after weight loss.	*N* = 55 Gender Women *n* = 45 Men *n* = 10 Mean BMI (kg/m^2^) Women 29 ± 6.2 Men 32 ± 9.3 Mean Age (Years) Woman 48 ± 10.2 Men 50 ± 10.3	Evolving Self View (ESV) Body Shape Questionnaire (BSQ) Multidimensional Health Locus of Control (MHLC) Weight Locus of Control (WLOC) Toronto Alexithymia Scale (TAS 20) Quality of Life survey (SF36v2)	The sample of women presented body image preoccupations, identifying themselves in’ I obese’ group for the ESV, 18–30 months after bariatric surgery.
Perdue et al. (44)	Evaluation of psychosocial adaptation in female patients undergoing bariatric surgery	*N* = 40 women Mean BMI (kg/m^2^): 28.31 ± 6.14 Mean Age (Years): 48 ± 9.97	Evolving Self View (ESV) Multidimensional Health Locus of Control (MHLC) Weight Locus of Control (WLOC) Toronto Alexithymia Scale (TAS 20) Body Shape Questionnaire (BSQ) Quality of Life survey (SF36v2).	Most of the sample still saw themselves as people with obesity, despite their weight loss. They had low scores in the vitality dimension and in the areas of social functioning and mental health, with difficulties in recognising their emotions.
Monpellier et al. (60)	Assessing the relationship between body image, depressive symptoms and weight loss in post-bariatric patients and evaluating whether there is a desire for body contouring surgery.	*N*= 590 Divided in 3 subgroups: 1. BCS group = body contouring surgery *N* = 65 Gender: women 61 Mean BMI (kg/m^2^): 28.5 ± 4.6 Mean Age (Years): 45.0 ± 11.4 2.D group = desire surgery *N* = 368 Gender: women 311 Mean BMI (kg/m^2^): 31.2 ± 5.4 Mean Age (Years): 47.1 ± 10.6 3.ND group = no desire surgery *N* = 157 Gender: women 107 Mean BMI (kg/m^2^): 31.9 ± 6.0	Body Shape Questionnaire (BSQ). Multidimensional Body-Self Relations Questionnaire- 121 Appearance Scales. Beck Depression Inventory II (BDI II).	Most of the patients had low scores on the appearance assessment and body image satisfaction scales and presented with depressive symptoms, showing signs of body contouring surgery.
Behrens et al. (61)	To observe the relationship between body image, percentage of weight loss (%EWL) and improvement of depressive symptoms.	*N* = 75 Gender Women 42 Men 33 Mean BMI (kg/m^2^): 36.8 ± 8 Mean Age (Years): 44.3 ± 11.5	Body image questionnaire (BIQ) Patient Health Questionnaire (PHQ-9)	The improvement in depressive symptoms was not directly related to the percentage of weight loss, but rather to the change in body image.
Pineda-García et al. (62)	Testing a self-care model that relates the sense of self efficacy, the perception of body image, obsessive-compulsive disorder and depression in people undergoing bariatric surgery.	*N* = 102 Gender Women 92 Men 10 Mean BMI (kg/m^2^) not reported in the study Mean Age (Years) Women 39.77 ± 10.05 Men 40.9 ± 5.64	Symptom checklist-90-revised (SCL-90-R) Self-care agency scale (ASA) Self efficacy test Body shape questionnaire (BSQ) Body image assesment for obesity (BIA-O)	The co-presence of positive levels of self-efficacy and positive levels of body satisfaction may be predictive for high self-care capacity.
Campedelli et al. (63)	To assess the role of body image in relation to body mass index and to observe the association with psychophysical well-being in a clinical sample of patients undergoing bariatric surgery.	*N* = 59 Gender Women 44 Men 15 Mean BMI (kg/m^2^): 41.96 ± 5.38 Mean Age (Years): 39.17 ± 10.11	Symptom checklist-90-revised (SCL-90-R) Body uneasiness test (BUT) Short form health survey (SF-12) Beck depression inventory II (BDI-II). Beck hopelessness scale (BHS)	Body image plays a more significant role than BMI in its association with psychological outcomes.
Bosc et al. (64)	Body image assessment using the Stunkard Figure Rating Scale and the Multidimensional Body Self Relations Questionnaire Appearance Scale before bariatric surgery and at regular intervals up to 5 years after surgery.	*N* = 61 Gender Women 47 Men 14 Mean BMI (kg/m^2^): 42.4 ± 7.9 Mean Age (Years): 45 ± 10	Multidimensional body-self relations questionnaire-appearance scale (MSBQR-AS). Stunkard figure rating scale.	Improvement in body image occurs in the first few months after surgery, but is not maintained for up to 5 years after surgery.

Importantly, the studies summarised in Table 2 rely primarily on self-report measures of body image and psychosocial variables. Accordingly, the discussion below explores how these findings may inform hypotheses regarding the potential involvement of body schema mechanisms in post-bariatric adaptation.

Within this field of literature on body image in the post-bariatric period, the studies conducted by Perdue and colleagues (43, 44) play a particularly important role, as they offer a deeper interpretative framework for understanding the mechanisms underlying the persistence of body dissatisfaction. Specifically, their findings show that, despite significant weight loss, many patients continue to perceive themselves as individuals with obesity, suggesting that their self-representation has not fully transitioned from “a person with obesity” to “a person formerly living with obesity.”

The identification of this failed transition was made possible through the use of a tool specifically developed by Perdue and colleagues, the *Evolving Self-View after Bariatric Surgery* (ESV) (43, 44). This instrument is designed to assess post-bariatric identity orientation by distinguishing between “I-obese” and “I-ex-obese” positions (the terms “obese” and “ex-obese” refer to the labels used in the original instrument), based on patterns of thinking, behaviour, and relational functioning rather than only on evaluations of physical appearance. Importantly, it should be noted that the ESV primarily assesses explicit self-representation rather than implicit body schema. Nevertheless, its findings may provide conceptually relevant observations for developing hypotheses regarding body schema updating after bariatric surgery. These findings suggest that, in the post-bariatric period, weight loss alone may be insufficient to modify deeply internalised body representations that continue to shape how individuals perceive themselves, act, and relate to others in the social context.

The failure to transition from the “I-obese” to the “I-ex-obese” observed in some patients after bariatric surgery may suggest a possible misalignment between actual bodily change and implicit bodily representations, although this interpretation remains speculative in the absence of direct measures of body schema, raising questions about the role of the body schema as a dynamic system of sensorimotor and postural integration.

Indeed, Pitron et al. (42), in a review, observes that the body schema exhibits long-term plasticity and adaptability, unlike body image, which can change rapidly. However, it is not yet known whether the two systems influence each other—that is, whether long-term changes in body image can affect the body schema and vice versa (42).

**Figure 2:**
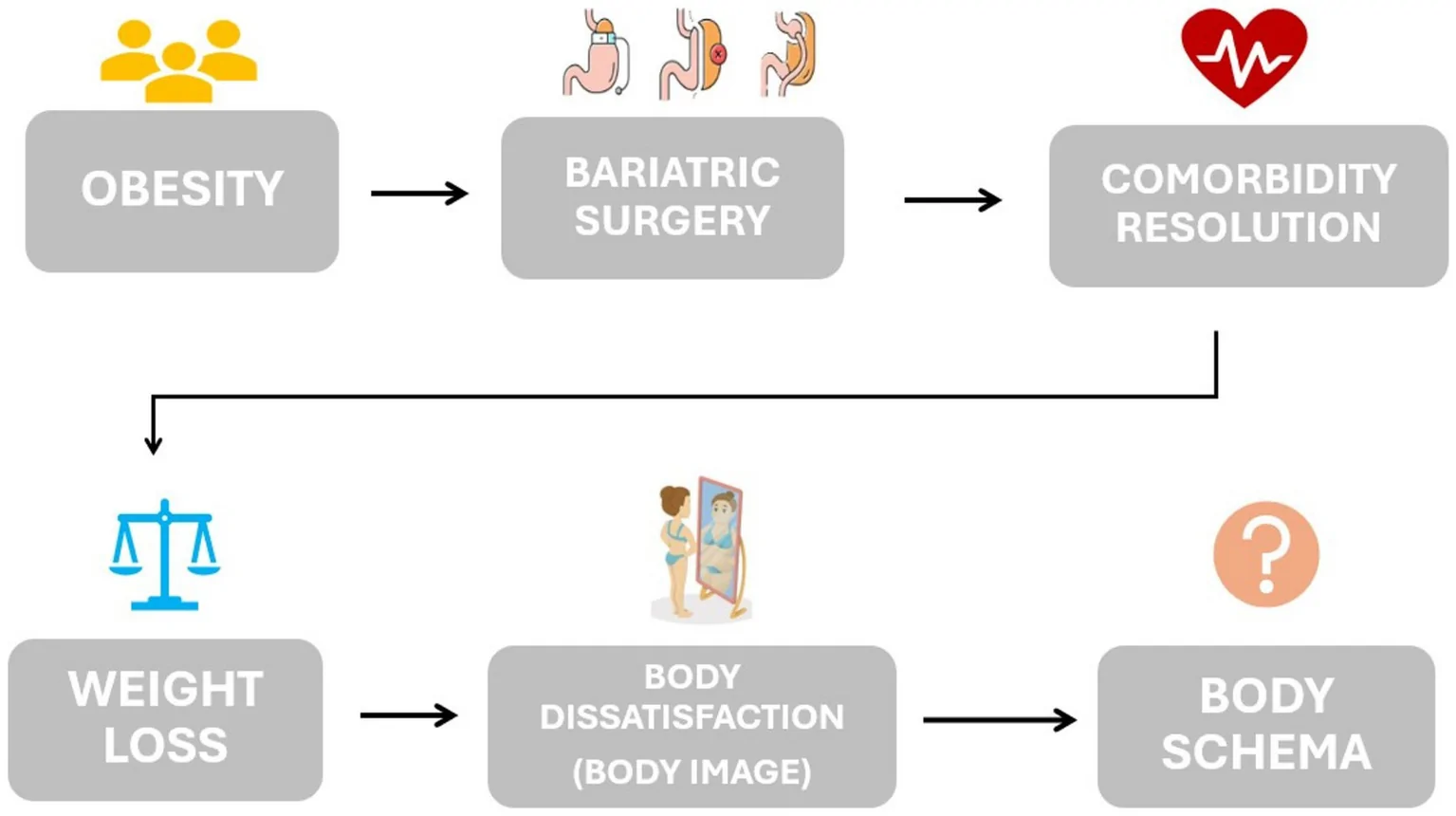
Schematic representation of the post-bariatric patient experience.

This distinction between the relatively rapid adaptation of body image and the potentially slower adaptation of body schema may help explain why substantial morphological changes do not necessarily translate into a corresponding update of implicit bodily representations following bariatric surgery. This raises an important question: why do some patients continue to perceive themselves as living with obesity despite substantial postoperative weight loss? (Figure 2).

From a neuroscientific perspective, body representation cannot be reduced to a single brain region or to primary somatosensory processing alone, but rather emerges from the activity of distributed networks integrating sensory, proprioceptive, interoceptive, motor, and spatial information, involving parietal, premotor, and insular regions. As emphasised by contemporary models of body representation, the body is therefore represented across multiple levels, supporting not only the perception of the body in space but also the sense of body continuity and identity over time (41).

In this framework, body schema can be understood as an implicit functional system oriented toward action and posture, which is constructed through repeated bodily experience and tends to maintain relative stability to ensure perceptual-motor coherence. This relative stability, however, may translate into limited flexibility in response to rapid and substantial body changes, such as those induced by bariatric surgery. Past body experiences, especially if prolonged, contribute to the construction of deep body representations that do not automatically update in response to a recent morphological change (46).

In this regard, the “allocentric lock” hypothesis proposed by Riva (47) offers a potentially relevant interpretative framework, although its direct role in post-bariatric populations remains to be specifically tested. According to this model, individuals with obesity may be more inclined to rely on an observer’s perspective (allocentric level) when retrieving body representations stored in long-term memory. As a result, they may remain anchored to memories of past events characterised by negative self-evaluation based on physical appearance, especially among individuals who have experienced weight-related stigma. This process may lead to the crystallisation of body representation, which persists despite substantial bodily changes following weight loss. Such persistence has been linked to amygdala functioning, as weight stigma can evoke negative emotional responses that are encoded in the amygdala and may contribute to mechanisms influencing body representation updating (47).

The persistence of these representations may be further reinforced by altered interoceptive and somatosensory processing observed in individuals with obesity (48–50). In this sense, the post-bariatric body, although profoundly modified morphologically, could continue to be experienced according to previous sensory and memory schemas (51–56).

From this perspective, the study by Propice et al. (57) further suggests that not only obesity itself, but also the history of bodily transformations, as in the case of weight cycling (frequent in obesity), can influence the stability of the body schema. In particular, the study highlights a direct link between whole-body morphological changes and alterations in body schema, suggesting that significant changes in body mass and size require continuous updating of the sensorimotor mechanisms underlying body adaptation. When such changes are marked or frequent, the ability to integrate the sensory information necessary to update body representation may be compromised (57).

Finally, long-term neuroimaging data following bariatric surgery indicate that, 2 years after surgery, the brain does not undergo global remodeling, but rather selective structural and functional remodeling (58). This evidence may be consistent with the hypothesis of a temporal misalignment between rapid body change and the slower neural reorganisation of deep body representations. Accordingly, the persistence of obesity-related self-representation may not be attributable solely to psychological resistance to change but may also reflect complex, gradual, and sometimes incomplete neural updating processes. Rather, based on indirect evidence, this persistence may also reflect a mismatch between rapid morphological change and the slower updating of implicit body representations. From this perspective, bariatric surgery appears to primarily modify the physical body, whereas the sensorimotor and embodied processes through which the body is experienced may require a longer period of adaptation. When internal body models have been shaped by many years of obesity, repeated stigmatization, and altered sensorimotor experiences, a sudden reduction in body mass may not be sufficient to update these well-established representations.

This conceptual framework raises an important clinical and theoretical question: how can post-bariatric care support not only weight loss and health improvement, but also the integration of a new body condition at the level of implicit body experience?

From a psychological perspective, these observations suggest the potential value of interventions targeting body image and bodily self-awareness to facilitate the integration of rapid postoperative bodily changes. A comprehensive understanding of post-bariatric adaptation cannot be limited to body image or body schema alone, but should also encompass neurocognitive and sensorimotor processes, as well as the individual’s history of body change, including repeated cycles of weight loss and regain. An integrated approach of this kind can offer psychologists and clinical professionals broader tools to help patients recognise and integrate their post-surgical bodies, going beyond simple physical modification. As a concept-driven narrative review, the selection of studies was guided by theoretical relevance rather than systematic criteria. Therefore, the review may be subject to selection bias and may not have captured all potentially relevant literature. This limitation should be considered when interpreting the proposed theoretical framework.

## 7 Conclusion

Bariatric surgery represents one of the most effective treatments for severe obesity, leading to substantial weight loss and significant improvements in metabolic health and obesity-related comorbidities. However, as highlighted in this narrative review, the profound morphological changes induced by surgery do not necessarily translate into a corresponding update in how the body is implicitly experienced and represented.

Distinguishing between body image and body schema provides a particularly useful conceptual framework for interpreting this apparent paradox. However, it is important to note that the involvement of body schema mechanisms in post-bariatric adaptation remains largely hypothetical, as most available studies have relied on explicit body image measures rather than direct assessments of implicit sensorimotor representations. Body image, understood as an explicit, conscious, and socio-emotional representation of the body, may undergo relatively rapid changes following weight loss. In contrast, body schema relies on implicit sensorimotor, proprioceptive, and interoceptive processes, supported by distributed neural circuits, which are shaped over time through long-term sensorimotor learning. When rapid changes in body mass occur, as is the case after bariatric surgery, these neurally entrenched representations may not fully update proportionally, resulting in a persistent discrepancy between the physical body and the body as represented at the explicit level. One possible explanation for this mismatch is that it may arise from constraints on the plasticity of body schema-related neural systems, particularly in individuals with a long history of obesity, weight stigma, altered sensorimotor experience, or repeated weight cycling. In this context, the persistence of obesity-related self-representations after surgery should not be understood merely as a form of psychological resistance or dissatisfaction with surgical outcomes, but rather as potentially reflecting a delayed or incomplete recalibration of implicit body representations. These considerations have important clinical implications. If bariatric surgery primarily acts on the physical body, post-operative care should extend beyond weight loss to address the implicit bodily processes that support action, posture, and bodily awareness. Current evidence supports the use of psychological approaches targeting body image, including cognitive-behavioural interventions, psychoeducation, and mindfulness-based strategies, which may facilitate psychological adjustment and body satisfaction following surgery. In addition, targeted interventions aimed at sensorimotor integration and bodily awareness, such as sensorimotor re-education, motor imagery, mirror exposure, and virtual reality-based approaches, may represent promising complementary strategies to facilitate the integration of rapid bodily changes within multidisciplinary bariatric follow-up. Although some evidence supports these interventions in relation to body image, their effectiveness in addressing body schema and implicit body representations in bariatric populations remains to be established. The present framework should therefore be viewed as a hypothesis-generating model intended to guide future empirical investigations rather than as a definitive explanation of post-bariatric adaptation. The processes underlying post-bariatric adaptation are likely multifactorial, involving not only psychological and sensorimotor mechanisms but also broader neurobiological factors. In this regard, neurological complications related to nutritional deficiencies have been reported after bariatric surgery, underscoring the importance of considering the complex interactions between metabolic, neurological, and psychological processes during postoperative adaptation (59).
